# Metaheuristic Algorithm and Laser Projection for Adjusting the Model of the Last Lower Surface to a Footprint

**DOI:** 10.3390/biomimetics9110699

**Published:** 2024-11-14

**Authors:** J. Apolinar Muñoz Rodríguez

**Affiliations:** Centro de Investigaciones en Óptica, A. C., Lomas del Bosque 115, Col. Comas del Campestre, León 37000, GTO, Mexico; munoza@cio.mx

**Keywords:** metaheuristic genetic algorithms, Bezier surface model, laser line scanning, last lower-surface adjustment

## Abstract

Nowadays, metaheuristic algorithms have been applied to optimize last lower-surface models. Also, the last lower-surface model has been adjusted through the computational algorithms to perform custom shoe lasts. Therefore, it is necessary to implement nature-inspired metaheuristic algorithms to perform the adjustment of last lower-surface model to the footprint topography. In this study, a metaheuristic genetic algorithm is implemented to adjust the last lower surface model to the footprint topography. The genetic algorithm is constructed through an objective function, which is defined through the last lower Bezier model and footprint topography, where a mean error function moves the last lower surface toward the footprint topography through the initial population. Also, the search space is deduced from the last lower surface and footprint topography. In this way, the genetic algorithm performs explorations and exploitations to optimize a Bezier surface model, which generates the adjusted last lower surface, where the surface is recovered via laser line scanning. Thus, the metaheuristic algorithm enhances the last lower-surface adjustment to improve the custom last manufacture. This contribution is elucidated by a discussion based on the proposed metaheuristic algorithm for surface model adjustment and the optimization methods implemented in recent years.

## 1. Introduction

Nowadays, metaheuristic optimization algorithms provide a powerful tool to perform surface modeling [1]. This mathematical modeling plays an important role in representing the last lower surface in footwear manufacturing [2], where the custom footwear manufacturing determines if the last lower surface fits the target footprint topography [3]. Also, a personalized last lower surface is adjusted by moving the last lower surface toward the desired footprint topography. Thus, a good contact between the footprint and the shoe insole is achieved. This kind of contact provides a good pressure distribution on the plantar surface, which supplies good body functionality [4]. Typically, the last lower surface adjustment is performed by modifying parameters from a constructed surface model [5]. In this way, an algorithm of modifications is implemented to adjust the surface model. Thus, the last lower adjustment is performed by means of surface model optimization and an algorithm of modifications, where the surface model optimization is performed by means of mathematical algorithms and surface data [6]. But the algorithm of modifications is carried out by changing morphological parameters and surface data in the surface model. 

In this way, the traditional last lower adjustment is implemented by constructing a surface model via optimization and applying an algorithm to perform modifications. For instance, a shoe last bottom model has been constructed by means of constraints in AutoCAD and an optimization of morphological parameters [7]. But the surface model modifications are performed by changing a set of morphological parameters, which modify the original surface. Also, the last bottom model has been built via CAD/CAM technologies by optimizing the parameters and regression function [5], where the surface model modifications are performed by changing the size of the morphological parameters to modify the original surface. In the same way, the last lower-surface model has been constructed through the Taguchi method by optimizing the parameters of a regression function [8]. But the surface model modifications are performed by changing the tolerance of the morphological parameters. Also, the last surface model has been implemented by optimizing the NURBS parameters [9], where the surface model modifications are carried out by changing the size of morphological parameters. In the same way, the last lower-surface model has been built via NURBS optimization [10], where surface model modifications are carried out via landmarks and a set of foot parameters. Moreover, the last lower-surface model has been constructed based on the principal component optimization [11,12], where the algorithm of modifications is carried out through the foot parameters. Furthermore, the insole surface model has been generated by means of a quadratic model based on the pressure points [13], where the surface model modifications are carried out through the medical images of the foot parameters. Additionally, the foot surface model has been constructed via NURBS based on landmarks [14], where the NURBS surface model is adjusted by employing the landmarks of the foot parameters.

Based on the above statements, it is established that the optimization algorithm performs an important role to accomplish the surface model. In this field, metaheuristic algorithms such as particle swarm, ant colony, simulated annealing and fuzzy logic have been implemented to optimize the surface model. For instance, a NURBS surface model has been optimized trough the particle swarm algorithm [15], where the particle swarm performs the optimization through an inertia equation and a particle velocity equation. In the same way, a NURBS surface model has been optimized via the ant colony algorithm [16], where the ant colony algorithm makes trajectories through a pheromone concentration to obtain the surface model parameters. Also, surface model optimization has been performed through the simulated annealing algorithm [17], where the simulated annealing decreases and increases an objective function by means of a small perturbation to determine the parameters. Additionally, a surface model optimization has been implemented via fuzzy logic [18], where the model parameters are deduced by means of a contactless scanning. Furthermore, the last lower-surface adjustment has been carried out by changing the morphological parameters [19,20], where the modifications are performed by correlating different surface models. Also, the last lower-surface modification has been performed based on a finite element method [21], where the adjustment is carried out by changing the morphological parameters. In the same way, the last lower adjustment has been performed via the Taguchy method by combining sets of the morphological parameters [18]. 

The above-mentioned methods perform the surface model optimization via artificial intelligence algorithms. However, these methods do not optimize the surface model parameters through the search space based on the surface data. For instance, simulated annealing, particle swarm, ant colony, and fuzzy logic algorithms do not generate the solution space based on the surface data. Therefore, additional procedures should be performed to achieve the optimal surface model. Also, the optimization is carried out through an objective function, which includes additional parameters to the surface model. This increases the number of iterations to optimize the surface model. Moreover, these methods do not include surface model adjustment. Therefore, the procedure to perform the adjustment of the last lower-surface model still needs research and computational development. Based on these statements, it is deduced that the adjustment of the last lower-surface model still represents a complicated task. To enhance this technology, it is necessary to implement a metaheuristic genetic algorithm to adjust the surface model toward the footprint topography. 

The proposed technique performs the adjustment of the last lower-surface model through a metaheuristic genetic algorithm and laser line projection. This metaheuristic algorithm provides a more suitable structure to perform the optimization of the adjusted last lower-surface model. For instance, the particle swarm optimization, ant colony, simulated annealing and fuzzy logic generate the initial population in random form [22,23,24,25]. But the next generation is generated based on the previous population. These two steps produce high error and dependence on the current solution. Instead, the metaheuristic algorithm provides an initial population based on surface data, and a flexible structure to test candidates. This procedure produces low error and the next generation is independent of the current solution. From these attributes, the iterations of the optimization are reduced. Also, the traditional algorithms employ additional equations to optimize the surface model. For instance, the particle swarm computes a position equation and velocity equation, ant colony and simulated annealing compute a probability function, and fuzzy logic computes a linguistic function [26,27,28,29]. This leads to producing smaller changes in the next solution and dependence on the current solution. On the other hand, the metaheuristic algorithm does not compute additional equations, and it can produce bigger and smaller changes through the mutation without any dependence. Based on these improvements, the metaheuristic genetic algorithm performs the optimization of the adjusted last lower-surface model. 

The surface modeling adjusts the last lower surface toward the footprint topography by means of the surface data, which are retrieved via laser line scanning. In this procedure, the last lower-surface model is generated by means of 5-th order Bezier basis functions and control points. Thus, the metaheuristic algorithm optimizes the last lower-surface model, which fits to the footprint topography through the surface data. To carry it out, the metaheuristic algorithm generates the search space from the last lower surface and the footprint topography. Based on the search space, the last lower surface is moved toward the footprint by means of a mean error function and the initial population, where the mean error function provides the difference between the last lower surface and the footprint topography. Then, explorations and exploitations are performed to accomplish the optimal control points, which provide the adjusted last lower surface. The last lower-surface modeling is carried out through a vision system, which consists of a CCD camera and a laser line. This arrangement is mounted on a slider device to perform laser line scanning, where the laser line is projected perpendicularly on the last lower surface and the CCD camera captures the laser line, which reflects the surface contour. In this way, the surface dimension is computed by means of the setup geometry and the laser line position. Then, the last lower-surface model is generated via Bezier basis functions, which are adjusted to the footprint topography based on the mean error function. Thus, the metaheuristic algorithm and laser line scanning enhances the accuracy of the adjustment of the last lower-surface model to the footprint topography. The contribution of the metaheuristic genetic algorithms is established based on the surface model adjustment, run time, and algorithm structure. To elucidate this contribution, a discussion is carried out based on the surface model adjustment of recent years.

This paper is organized as follows: the metaheuristic algorithm to perform the 5th-order Bezier surface model is explained in Section 2.1, the surface contouring via laser line scanning is described in Section 2.2, the adjustment of the last lower-surface model to the footprint is performed in Section 3, and the discussion of the contribution of the adjusted surface model is given in Section 4. 

## 2. Materials and Methods

### 2.1. Bezier Surface Modeling via Metaheuristic Algorithm

**Figure 1:**
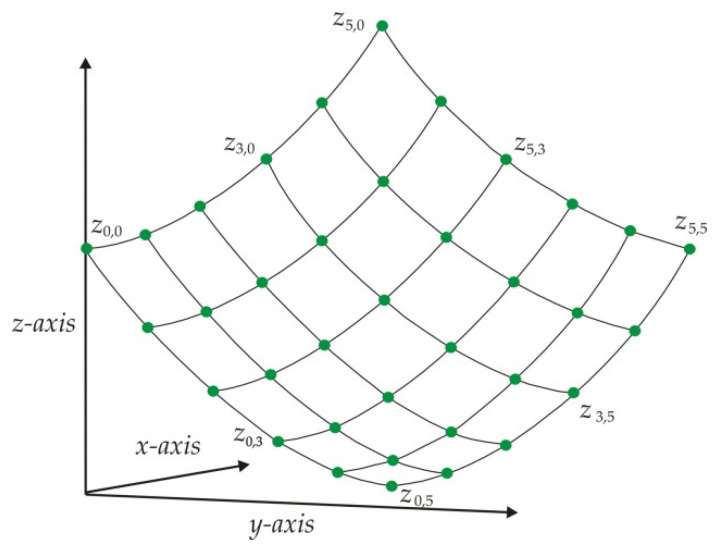
Surface points to construct a 5th-order Bezier surface model.

The last lower-surface model is built through the 5th-order Bezier surface model, which is optimized by a metaheuristic algorithm. To construct the Beszier surface model, the last lower surface is scanned via laser line to retrieve the surface data. Then, the Bezier surface model is generated by means of the surface points *z_i_*_,*j*_, which are shown in Figure 1. In this figure, the coordinates (*x_i_*_,*j*_, *y_i_*_,*j*_) represent the surface position on the *x*-axis and *y*-axis, but the sub-indices (*i*, *j*) represent the number of the surface points in the *x* and *y* direction, respectively. In this way, the 5th-order Bezier surface is built through the surface points (*z*_0,0_, *z*_1,0_, …, *z*_5,0_, …, *z*_5,5_). From these surface data, the Bezier surface is generated based on the control points *P_i_*_,j_ by means of the following expressions:

(1) $$S_{r,t}(u,v)=\sum_{i=0}^{i=5}\sum_{j=0}^{j=5}\frac{5!5!}{(5-i)!i!(5-j)!j!}{\left(1-u\right)}^{5-i}{\left(1-v\right)}^{5-j}u^{i}v^{j}P_{5r+i,5t+j},\;\;\;u,v\in [0,1],$$

$$u_{i,j}=\frac{\left(x_{5r+i,j}-x_{5r,j}\right)}{\left({x_{5(r+1)}}_{,j}-x_{5r,j}\right)},\;\;\;v_{i,j}=\frac{\left(y_{i,5t+j}-y_{i,5t}\right)}{\left(y_{i,5(t+1)}-y_{i,5t}\right)}.$$

In this equation, the control points *P*_5*r*+*i*,5*t*+*j*_ move the Bezier surface *S_r_*_,*t*_(*u*,*v*) toward the surface points *z*_5*r*+*i*,5*t*+*j*_, where the sub-indices (*r*, *t*) represent a surface region. But the parametric values (*u*, *v*) are defined on the *x*-axis and *y*-axis, respectively.

Based on these statements, the last lower-surface model is represented by the Bezier surfaces *S*_0,0_(*u*,*v*), *S*_1,0_(*u*,*v*), *S*_1,0_(*u*,*v*), and *S*_1,1_(*u*,*v*), …, *S_N_*_,*M*_(*u*,*v*), where *N* and *M* are the number of Bezier surfaces in the *x*-direction and *y*-direction, respectively. In this way, the Bezier surface *S_r_*_,*t*_(*u*,*v*) is generated by means of the control points *P*_5*r*+*i*,5*t*+*j*_. To compute the control points, the next equation is obtained from the Bezier surface Equation (1).

(2) $$S_{r,t}(u,v)=B_{0,0}(u,v)P_{5r+0,5t+0}+B_{0,1}(u,v)P_{5r+0,5t+1}+B_{0,2}(u,v)P_{5r+0,5t+2}+,\ldots \ldots ,+B_{5,5}(u,v)P_{5r+5,5t+5}.$$

For this equation, *B_i_*_,*j*_(*u*,*v*) = 5!5!(1 − *u*)^5−*i*^(1 − *v*)^5−*j*^*u^i^v^j^*/(5 − *i*)!*i*!(5 − *j*)!*j*!, and the control points are defined based on the weights W_5*r*+*i*,5*t*+*j*_ by the expression *P*_5*r*+*i*,5*t*+*j*_ = W_5*r*+*i*,5*t*+*j*_ *z*_5*r*+*i*,5*t*+*j*_. Thus, the parametric values (*u_i_*_,*j*_, *v_i_*_,*j*_) and the surface points *z_i_*_,*j*_, are substituted in Equation (2) to obtain the next equation system:

(3) $$\left[\begin{array}{l} S_{r,t}(u_{0,0},v_{0,0})\\ S_{r,t}(u_{0,0},v_{0,1})\\ S_{r,t}(u_{0,0},v_{0,2})\\ S_{r,t}(u_{5,5},v_{5,5}) \end{array}\right]=\left[\begin{array}{l} B_{0,0}(u_{0,0},v_{0,0})+B_{0,1}(u_{0,0},v_{0,0})+B_{0,2}(u_{0,0},v_{0,0})+\ldots +B_{5,5}(u_{0,0},v_{0,0})\\ B_{0,0}(u_{0,0},v_{0,1})+B_{0,1}(u_{0,0},v_{0,1})+B_{0,2}(u_{0,0},v_{0,1})+\ldots +B_{5,5}(u_{0,0},v_{0,1})\\ B_{0,0}(u_{0,0},v_{0,2})+B_{0,1}(u_{0,2},v_{0,0})+B_{0,2}(u_{0,2},v_{0,0})+\ldots +B_{5,5}(u_{0,0},v_{0,2})\\ \vdots \;\;\;\;\;\vdots \;\;\;\;\;\;\vdots \;\\ B_{0,0}(u_{5,5},v_{5,5})+B_{0,1}(u_{5,5},v_{5,5})+B_{0,2}(u_{5,5},v_{5,5})+\ldots +B_{5,5}(u_{5,5},v_{5,5}) \end{array}\right]\left[\begin{array}{l} W_{5r+0,5t+0},z_{5r+0,5t+0}\\ W_{5r+0,5t+1},z_{5r+0,5t+1}\\ W_{5r+0,5t+2},z_{5r+0,5t+2}\\ \vdots \\ W_{5r+5,5t+5},z_{5r+5,5t+5} \end{array}\right].$$

For this equation system, the Bezier surface *S_r_*_,*t*_(*u_i_*_,*j*_,*v_i_*_,*j*_) is replaced by the surface data *z*_5*r*+*i*,5*t*+*j*_, and the values (*u_i_*_,*j*_, *v_i_*_,*j*_) are computed through the expressions given in Equation (1). Thus, the weights W_5*r*+*i*,5*t*+*j*_ are computed through a metaheuristic algorithm by means of the following steps. 

In the first step, the search space and the initial population are determined based on the last lower surface and the footprint topography. To carry it out, the last lower surface is overlapped onto the footprint topography. From this overlapping, the mean error between the last lower surface *z_i_*_,*j*_ and the footprint topography *h_i_*_,*j*_ is computed by the following expression:

(4) $$E_{r,t}=\frac{1}{36}\sum_{i=0}^{5}\sum_{j=0}^{5}(z_{5r+i,5t+j}-h_{5r+i,5t+j}).$$

Then, the surface points *z*_5*r*+*i*,5*t*+*j*_ are moved toward the footprint topography by computing the expression *z*_5*r*+*i*,5*t*+*j*_ = *z*_5*r*+*i*,5*t*+*j*_ − *E_r_*_,*t*_, for *i* = 0, 1, 2, …, 5 and for *j* = 0, 1, 2, …, 5. This procedure moves the last lower surface toward the footprint topography by means of the mean error function. Thus, if *E_r_*_,*t*_ > 0, the surface *z*_5*r*+i_,_5*t*+j_ is moved down. But, if *E_r_*_,*t*_ < 0, the surface *z*_5*r*+i,5*t*+j_ is moved up. Additionally, the border surface points (*z*_5*r*+5,5*t*+1_, *z*_5*r*+5,5*t*+2_, *z*_5*r*+5,5*t*+3_, *z*_5*r*+5,5*t*+4_) and (*z*_5*r*+1,5*t*+5_, *z*_5*r*+2,5*t*+5_, *z*_5*r*+3,5*t*+5_, *z*_5*r*+4,5*t*+5_) are computed by the expressions *z*_5*r+5,*j*_ = (*z*_5*r+3,*j*_ + *z*_5*r+6,*j*_)/2 and *z_i_*_,5*t_ = *(z_i_*_,5*t+4_ + *z_i_*_,5*t+6_)/2 to obtain continuity *G*^1^. Then, the initial population is generated from the maximum and minimum of each weight. To determine the initial population, the Bezier surface *S_r_*_,*t*_(*u_i_*_,*j*_,*v_i_*_,*j*_) Equation (1) is computed by substituting W_5*r*+*i*,5*t*+*j*_ = 1 and *P*_5*r*+*i*,5*t*+*j*_ = *z*_5*r*+*i*,5*t*+*j*_. Thus, if *S_r_*_,*t*_(*u_i_*_,*j*_, *v_i_*_,*j*_) is over the surface *z*_5*r*+*i*,5*t*+*j*_, the maximum is equal to 1, and the minimum is given by [*z*_5*r*+*i*,5*t*+*j*_ − 3 * abs(*z*_5*r*+*i*,5*t*+*j*_ − *h*_5*r*+*i*,5*t*+*j*_)]/*z*_5*r*+*i*,5*t*+*j*_. But, if *S_r_*_,*t*_(*u_i_*_,*j*_, *v_i_*_,*j*_) is under *z*_5*r*+*i*,5*t*+*j*_, the minimum is equal is 1 and the maximum is given by [*z*_5*r*+*i*,5*t*+*j*_ + 3*abs(*z*_5*r*+*i*,5*t*+*j*_ − *h*_5*r*+*i*,5*t*+*j*_)]/*z*_5*r*+*i*,5*t*+*j*_. Thus, the search space has been determined for each weight W_5*r*+*i*,5*t*+*j*_. From this search space, four values are randomly taken to obtain the initial population for each weight. These values are defined as the parents (P_1,*k*_, P_2,*k*_, P_3,*k*_, P_4,*k*_), which represent the initial population. In this case, the *k*-sub-index represents the generation number. This initial population produces an adjusted Bezier surface based on the mean error function.

The second step computes the *k*-generation children via crossover by means of explorations and exploitations [30]. This procedure computes two children inside parents and one child outside parents. To carry it out, the children (C_1,*k*_, C_2,*k*_) and (C_4,*k*_, C_5,*k*_) are computed via exploration from the parents (P_1,*k*_, P_2,*k*_) and (P_3,*k*_, P_4,*k*_), respectively. Then, the children (C_3,*k*_, C_6,*k*_) are computed via exploitation. In this way, the current children are calculated through the following expressions:

(5) $$C_{1,k}=\;0.5\left[\left(P_{1,k}+P_{2,k}\right)+\beta \left|P_{1,k}-P_{2,k}\right|\right],$$

(6) $$C_{2,k}=\;0.5\left[\left(P_{1,k}+P_{2,k}\right)-\beta \left|P_{1,k}-P_{2,k}\right|\right],$$

(7) $$C_{4,k}=\;0.5\left[\left(P_{4,k}+P_{3,k}\right)+\beta \left|P_{4,k}-P_{3,k}\right|\right],$$

(8) $$C_{5,k}=\;0.5\left[\left(P_{4,k}+P_{3,k}\right)-\beta \left|P_{4,k}-P_{3,k}\right|\right],$$

(9) $$C_{3,k}=P_{0,k}+\beta \left|P_{1,k}-P_{0,k}\right|,$$

(10) $$C_{6,k}=P_{4,k}+\beta \left|P_{5,k}-P_{4,k}\right|.$$

For these equations, P_0,*k*_ and P_5,*k*_ are the minimum and maximum of each weight. Additionally, the parameter *β* is calculated by means of the factor *α*, which is taken in random form from the interval between 0 and 1. In this way, *β* = (2*α*)^1/2^ if *α* > 0.5; otherwise, *β* = [2(1 − *α*)]^1/2^. Thus, Equations (5)–(8) compute the children inside parents, and Equations (9) and (10) compute the children outside parents. Thus, the *k*-generation children are computed. 

The third step computes the fitness of the Bezier surfaces *S_r_*_,*t*_(*u_i_*_,*j*_,*v_i_*_,*j*_) by means of an objective function, which is described by the following expression:

(11) $$O_{r,t}=\min\left\{\sqrt{\frac{1}{36}\sum_{i=0}^{i=5}\sum_{j=0}^{j=5}{\left[S_{r,t}(u_{i,j},v_{i,j})-z_{5r+i,5t+j}\right]}^{2}}\right\}.$$

This equation computes the fitness by means of the last lower surface *z*_5*r*+*i*,5*t*+*j*_ and the Bezier surface *S_r_*_,*t*_(*u_i_*_,*j*_,*v_i_*_,*j*_). The fourth step takes the best current parents and children to determine the (*k* + 1)-generation parents. Thus, the parent P_1,*k*+1_, is obtained from (P_1,*k*_, P_2,*k*_), the parent P_3,*k*+1_ is collected from (P_3,*k*_, P_4,*k*_), the parent P_2,*k*+1_ is taken from (C_1,*k*_, C_2,*k*_, C_3,*k*_) and the parent P_4,*k*+1_ is chosen from (C_4,*k*_, C_5,*k*_, C_6,*k*_). 

The fifth step mutates one parent and one weight to avoid a local minimum. In this way, a new parent replaces the worst parent to compute the fitness via Equation (11). If the new parent improves the fitness, it replaces the worst parent. Otherwise, the mutation is not carried out. Also, a new weight replaces a weight from a parent, which is selected in random form, and the fitness is computed via Equation (11). Thus, if the new weight improves the fitness, it replaces the selected weight. Otherwise, the selected weight is not mutated. Thus, the (*k* + 1)-generation parents have been completed. Also, Equations (5)–(10) are computed to obtain the (*k* + 1)-generation children. Thus, the (*k* + 1)-generation population is obtained. The steps two to five are iteratively computed until finding the weights that minimize the objective function, in Equation (11). Then, the mean error function Equation (4) is computed for *r* = 1, 2, 3, …, *N* and *t* = 1, 2, 3, …, *M*. In this case, the surface (*z*_5*r*+*i*,5*t*+*j*_) is replaced by *S_r_*_,t_(*u_i_*_,*j*_, *v_i_*_,*j*_) in Equation (4). Thus, if the absolute value of the mean error is more than a tolerance, go to compute step one to step five. But if the absolute value of the mean error is less than the tolerance, the metaheuristic algorithm has finished. 

**Figure 2:**
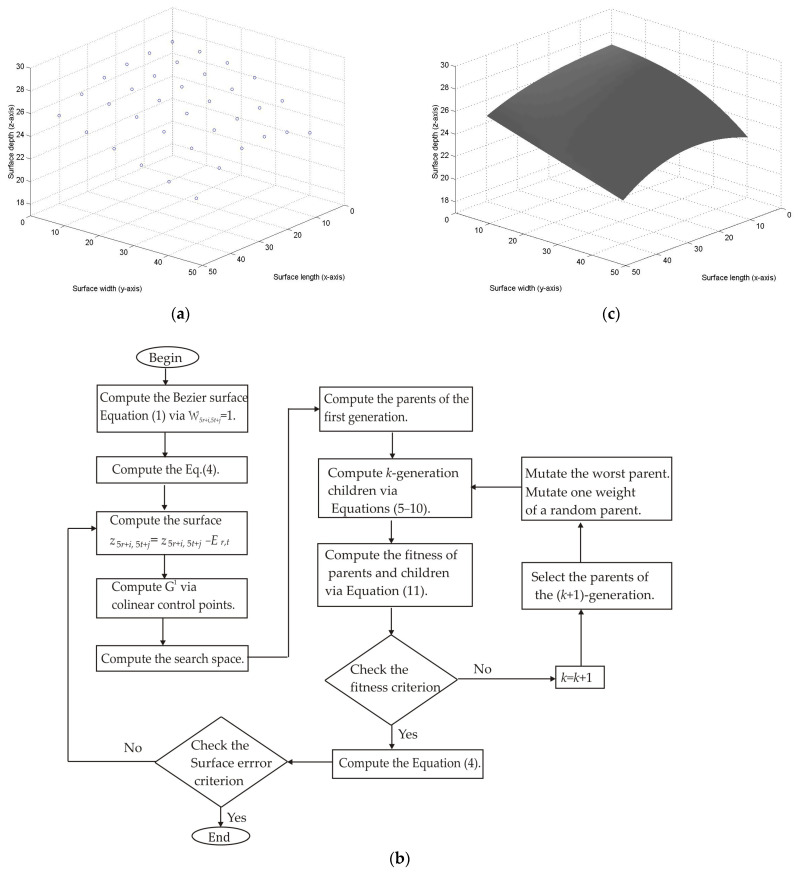
(**a**) Surface points to construct a Bezier surface. (**b**) Flowchart to perform metaheuristic algorithm for optimization of the control points of the Bezier surface model. (**c**) Bezier surface generated via control points optimized via metaheuristic algorithm.

To describe the steps of the metaheuristic algorithm, the Bezier surface *S*_0,0_(*u*, *v*) is computed from the surface points shown in Figure 2a. In this procedure, the control points (*P*_0,0_, *P*_0,1_, *P*_0,2_, …, *P*_5,5_) are computed through the genetic algorithm described in the flowchart shown in Figure 2b. For the Bezier surface *S*_0,0_(*u*, *v*), the control points *P*_0,0_ = 1, *P*_5,0_ = 1, *P*_0,5_ = 1, and *P*_5,5_ = 1 are provided by the Bezier surface Equation (1). Additionally, the control points (*P*_5,1_, *P*_5,2_, *P*_5,3_, *P*_5,4_) and (*P*_1,5_, *P*_2,5_, *P*_3,5_, *P*_4,5_) are computed by the expressions *P*_5*r+5,*j*_ = (*P*_5*r+3,*j*_ + *P*_5*r+6,*j*_)/2 and *P_i_*_,5*t_ = (*P_i_*_,5*t+4_ + *P_i_*_,5*t+6_)/2 to provide continuity *G*^1^. Based on these statements, the first step computes the mean error *E_r_*,*_t_* between the last lower surface and the footprint topography. Then, the last lower-surface points are computed by the expression *z*_5*r*+i,5*t*+j_ = *z*_5*r*+i,5*t*+j_ − *E_r_*_,*t*_. Also, the surface points (*z*_5*r*+5,5*t*+1_, *z*_5*r*+5,5*t*+2_, *z*_5*r*+5,5*t*+3_, *z*_5*r*+5,5*t*+4_) are computed via *z*_5*r+5,*j*_ = (*z*_5*r+3,*j*_ + *z*_5*r+6,*j*_)/2 to provide continuity *G*^1^. In the same way, the surface points (*z*_5*r*+1,5*t*+5_, *z*_5*r*+2,5*t*+5_, *z*_5*r*+3,5*t*+5_, *z*_5*r*+4,5*t*+5_) are computed by the expression *z_i_*_,5*t_ = *(z_i_*_,5*t+4_ + *z_i_*_5*t+6_)/2. Thus, the last lower-surface points are moved toward the footprint. Then, the initial population is generated from the maximum and minimum of each weight. To do so, the surface *S_r_*_,*t*_(*u_i_*_,*j*_,*v_i_*_,*j*_) Equation (1) is computed by substituting W_5*r*+*i*,5*t*+*j*_ = 1 and *P*_5*r*+*i*,5*t*+*j*_ = *z*_5*r*+*i*,5*t*+*j*_. Thus, if *S_r_*_,*t*_(*u_i_*_,*j*_, *v_i_*_,*j*_) > *z*_5*r*+*i*,5*t*+*j*_, the maximum is equal to 1, and the minimum is determined by the expression [*z*_5*r*+*i*,5*t*+*j*_ − 3*abs(*z*_5*r*+*i*,5*t*+*j*_ − *h*_5*r*+*i*,5*t*+*j*_)]/*z*_5*r*+*i*,5*t*+*j*_. But, if the surface *S_r_*_,*t*_(*u_i_*_,*j*_, *v_i_*_,*j*_) < *z*_5*r*+*i*,5*t*+*j*_, the minimum is equal to 1 and the maximum is given by the expression [*z*_5*r*+*i*,5*t*+*j*_ + 3*abs(*z*_5*r*+*i*,5*t*+*j*_ − *h*_5*r*+*i*,5*t*+*j*_)]/*z*_5*r*+*i*,5*t*+*j*_. From this search space, four values are randomly taken to determine the first parents (P_1,*k*_, P_2,*k*_, P_3,*k*_, P_4,*k*_), which represent the initial population of each control point. These first parents are shown in Table 1, where the first column depicts the control point to be optimized and the parents are shown in the second to fifth column. This initial population generates a Bezier surface moved toward the footprint topography. Then, the second step computes the current children via crossover, where the children (C_1,*k*_, C_2,*k*_, C_3,*k*_, C_4,*k*_, C_5,*k*_, C_6,*k*_) are computed via Equations (5)–(10). These children are shown in the sixth to eleventh column of Table 1. Next, the third step computes the fitness Equation (11) by employing the surface *S_r_*_,*t*_(*u_i_*_,*j*_, *v_i_*_,*j*_) and *z*_5*r*+*i*,5*t*+*j*_. This fitness is indicated in row seventeen. The fitness results show that the initial population produces a low error. Then, the fourth step chooses the (*k* + 1)-generation parents from the best current parents and children. In this way, the parent P_1,_*_k_*_+1_ is taken from (P_1,*k*_, P_2,*k*_), the parent P_3,*k*+1_ is collected from (P_3,*k*_, P_4,*k*_), the parent P_2,*k*+1_ is taken from (C_1,*k*_, C_2,*k*_, C_3,*k*_) and the parent P_4,*k*+1_ is chosen from (C_4,*k*_, C_5,*k*_, C_6,*k*_). Thus, P_1,2_ = P_1,1_, P_3,2_ = P_4,1_, P_2,2_ = C_3,1_, and P_4,2_ = C_5,1_. The fifth step takes the worst parent P_4,2_ to perform a mutation. 

To do this, a new parent replaces the worst parent to compute the fitness Equation (11). In this case, the new parent enhances the fitness; therefore, the new parent replaces the patent P_4,2_. Then, the parent P_3,2_ is randomly designated to mutate the weight W_2,2_, which is chosen in random form. To carry it out*,* a new weight replaces the weight W_2,2_ to calculate the fitness in Equation (11). In this case, the new weight does not improve the fitness. Therefore, the weight is not mutated. Then, the second step computes Equations (5)–(10) to obtain the (*k* + 1)-generation children. Also, the fitness is computed via Equation (11). The second generation population is shown in Table 2. The procedure to compute the (*k* + 1)-generation population is repeated until the objective function Equation (11) is minimized. From this procedure, the optimal weights are obtained to determine the control points, which are shown in the twelfth column of Table 2. These control points *P_i_*_,*j*_ = W*_i_*_,*j*_*z_i_*_,*j*_ are employed to generate the Bezier surface *S*_0,0_(*u*, *v*), shown in Figure 2c, where the control points (*P*_5,1_, *P*_5,2_, *P*_5,3_, *P*_5,4_) and (*P*_1,5_, *P*_2,5_, *P*_3,5_, *P*_4,5_) are computed by the expressions *P*_5*r+5,*j*_ = (*P*_5*r+31,*j*_ + *P*_5*r+6,*j*_)/2 and *P_i_*_,5*t_ = *(P_i_*_,5*t+4_ + *P_i_*_5*t+6_)/2 to provide continuity G^1^. 

In the same way, the Bezier surfaces *S*_0,1_(*u*,*v*), *S*_1,0_(*u*,*v*), …, *S*_N,M_(*u*,*v*) are computed to obtain the complete surface. Then, the absolute value of the mean error Equation (4) is computed to determine the difference between the Bezier surface and the footprint for *r* = 0, 1, 2, 3, …, *N* and *t* = 1, 2, 3, …, *M*. Thus, if the absolute mean error is more than a tolerance, the procedure from step one to step five is repeated. But, if the absolute value of the mean error is less than the tolerance, the metaheuristic algorithm has finished. The optical arrangement to compute the three-dimensional surface is described in Section 2.2. 

### 2.2. Three-Dimensional Surface Recovering via Laser Line Scanning

**Figure 3:**
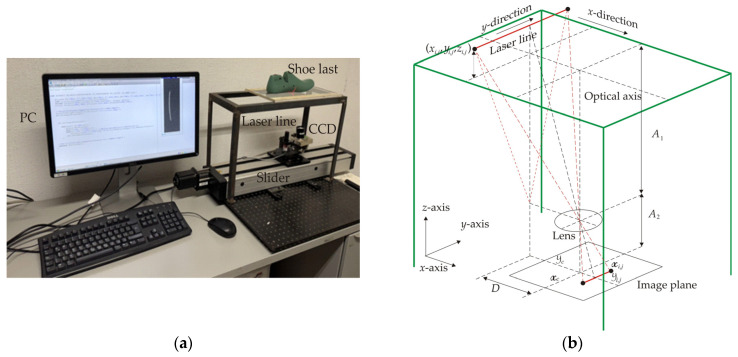
(**a**) Vision system to retrieve the last lower surface via laser line projection. (**b**) Vision system geometry to determine surface topography via laser line scanning.

The last lower surface and the footprint topography are recovered via laser line scanning through the vision system shown in Figure 3a. This optical arrangement consists of a laser line, a CCD array, a slider device and a PC computer. The laser line and the CCD camera are mounted on the slider device, which moves the setup horizontally via control software. In this arrangement, the *x*-axis represents the horizontal axis, the *y*-axis represents the vertical axis, and the z-axis depicts the surface height. Thus, the last lower surface is placed on the glass area, which represents the *x*-*y* plane, and the surface height is parallel to the *z*-axis. The geometry of the optical arrangement is sketched in Figure 3b, where the laser line is projected perpendicularly to the surface, and the CCD camera is placed at a distance *D* from the laser line. Based on this geometry, the transverse section of the laser line is placed in the *x*-direction, but, the longitudinal section of the laser line is depicted in the *y*-direction. These directions are indicated on the glass surface area in Figure 3b, where a surface point is represented by the coordinates (*x_i_*_,*j*_, *y_i_*_,*j*_, *z_i_*_,*j*_). In this case, the height between the surface and the glass is represented by *z_i_*_,*j*_. 

The distance between the glass and the camera lens is indicated by *A*_1_, but the length between the lens and the CCD array is represented by *A*_2_. The laser coordinates in the CCD camera are indicated by (x*_i_*_,*j*_, y*_i_*_,*j*_). The image center is given by the coordinates (x*_c_*, y*_c_*) and the pixel size is denoted by the symbol *η*. From the geometry shown in Figure 3b, the surface height *z_i_*_,*j*_ and the surface width *y_i_*_,*j*_ are determined by means of the following equations:

(12) $$z_{i,j}=\frac{A_{1}D}{\eta (x_{i,j}-x_{c})}-A_{2}.$$

(13) $$y_{i,j}=\frac{\eta (y_{i,j}-y_{c})(A_{2}+z_{i,j})}{A_{1}}+\eta y_{c}.$$

The surface length *x_i_*_,*j*_ is obtained from the laser line position, which is provided by the slider device, but the surface depth *z_i_*_,*j*_ and the surface width *y_i_*_,*j*_ are computed by means of the parameters (x*_c_*, y*_c_*, *η*, *A*_1_, *A*_1_, *D*). These parameters are computed through a genetic algorithm, which computes the objective function via Equations (12) and (13). To do so, the genetic algorithm computes the next five steps.

The first step computes the first population from the research space of each parameter. In this procedure, the maximum and minimum of the parameters (*x_c_*, *y*_c_, *η*) are obtained through the image size data, but the maximum and minimum of the parameters (*A*_1_, *A*_2_, *D*) are deduced from the setup geometry. In this way, the lens focus is established as the lens ratio of the lens. Also, the minimum *A*_1_ is defined as the lens ratio multiplied by 1.8 and the maximum *A*_1_ is established as the lens ratio multiplied by 3.8. In the same way, the minimum *A*_2_ is defined as the maximum *A*_1_, but, the maximum *A*_2_ is established as the maximum *A*_1_ multiplied by 10. Moreover, the minimum *D* is defined as the maximum *A*_1_, but, the maximum *D* is established as the maximum *A*_1_ multiplied by 8. Thus, the research space has been accomplished. Then, the parents (P_1,*k*_, P_2,*k*_, P_3,*k*_, P_4,*k*_) are randomly taken from the maximum and minimum of each parameter. Thus, for each parameter (*x_c_*, *y_c_*, *η*, *A*_1_, *A*_2_), four values are collected, and they represent the initial population. The second step computes Equations (5)–(10) to obtain the children (C_1,*k*_, C_2,*k*_, C_3,*k*_, C_4,*k*_, C_5,*k*_, C_6,*k*_). The third step determines the fitness through an objective function based on the surface height *z_i_*_,*j*_ and surface width *y_i_*_,*j*_ by means of the following equations:

(14) $$FO_{1}=\min\left\{\frac{1}{MxN}{\sum_{i=0}^{N}\sum_{j=0}^{M}\left[(z_{i,j}-z_{0,j})-\frac{A_{1}D}{\eta (x_{i,j}-x_{c})}+A_{2}+\frac{A_{1}D}{\eta (x_{0,j}-x_{c})}-A_{2}\right]}^{2}\right\},$$

(15) $$FO_{2}=\min\left\{\frac{1}{MxN}{\sum_{i=0}^{N}\sum_{j=0}^{M}\left[(y_{i,j}-y_{i,0})-\frac{\eta (y_{i,j}-y_{c})(A_{2}+z_{i,j})}{A_{1}}-\eta y_{c}+\frac{\eta (y_{i,0}-y_{c})(A_{2}+z_{i,0})}{A_{1}}+\eta y_{c}\right]}^{2}\right\}.$$

From these equations, the fitness is determined by the expression *FO* = (*FO*_1_ + *FO*_2_)/2, where (*z_i_*_,*j*_ − *z_i_*_,0_) and (*y_i_*_,*j*_ − *y_i_*_,0_) are known. The fourth step determines the (*k* + 1)-generation parents through the fitness, where P_1,*k*+1_ and P_3,*k*+1_ are taken from (P_1,*k*_, P_2,*k*_) and (P_3,*k*_, P_4,*k*_), respectively. But P_2,*k*+1_ and P_4,*k*+1_ are selected from (C_1,*k*_, C_2,*k*_, C_3,*k*_) and (C_4,*k*_, C_5,*k*_, C_6,*k*_), respectively. The fifth step changes the worst parent for a new parent, which is randomly selected from the solution space to compute the fitness. Thus, if the new parent improves the fitness, the new parent replaces the worst parent. Otherwise, the worst parent is not mutated. Also, a random parameter is changed to compute the fitness. If the new parameter enhances the fitness, the parameter is mutated. Otherwise, the parameter is not mutated. Thus*,* the (*k* + 1)-generation parents have been obtained. Based on these parents, the (*k* + 1)-generation children are computed via Equations (5)–(10), and the fitness is computed via Equations (14) and (15). Thus, the (*k* + 1)-generation population is achieved. The process to obtain the (*k* + 1)-generation population is iteratively performed until obtaining the parameters (*x_c_*, *y_c_*, *η*, *A*_1_, *A*_2_) that minimize Equations (14) and (15). 

The laser line coordinates are obtained from the maximum intensity in *x*-axis [31]. To compute the maximum intensity, a Bezier curve is fitted from the laser line pixels on the *x*-axis by means of the following equations:

(16) $$x(u)=\sum_{i=0}^{N}C_{i}{\left(1-u\right)}^{N-i}u^{i}x_{i,j},\;\;\;C_{i}=C_{i-1}(N+1-i)/i,\;\;\;C_{0}=1,\;\;\;0\leq u\leq 1.$$

(17) $$I(u)=\sum_{i=0}^{N}C_{i}{\left(1-u\right)}^{N-i}u^{i}I_{i,j},\;\;\;C_{i}=C_{i-1}(N+1-i)/i,\;\;\;C_{0}=1,\;\;\;0\leq u\leq 1.$$

In Equation (16), x*_i_*_,*j*_ indicates the laser line pixel position on the *x*-axis and *N* represents the number of laser line pixels; *I_i_*_,*j*_ depicts the pixel intensity in Equation (17). Additionally, the sub-indices (*i*, *j*) provide the laser line pixel number on the *x*-axis and *y*-axis, respectively. To perform the Bezier fitting, *I_i_*_,*j*_ is substituted in Equation (17) to obtain a concave curve *I*(*u*), whose second derivative *I*″(*u*) is positive in the interval 0 ≤ *u* ≤ 1. Thus, the maximum intensity is computed through the first derivative *I*′(*u*) = 0. In this case, the Bisection method computes the value *u* to find the derivative *I*′(*u*) = 0. Thus, *u* is replaced in Equation (16) to compute x*_i_*_,*j*_ = x(*u*), which represents the laser line position on the *x*-axis. The position y*_i_*_,*j*_ is obtained from the row number of the laser line image. The laser line edges y*_i_*_,0_ and y*_i_*_,*m*_ are determined by computing the first derivative on the *y*-axis. To determine the laser line coordinates (x*_i_*_,*j*_, y*_i_*_,*j*_), the CCD camera captures the laser line, and Equations (16) and (17) are computed, respectively. Then, the surface depth *z_i_*_,*j*_ is computed by replacing x*_i_*_,*j*_ in Equation (12) and the surface width *y_i_*_,*j*_ is computed by substituting y*_i_*_,*j*_ in Equation (13). The surface length coordinate on the *x*-axis is provided by the slider device. Thus, the three-dimensional surface topography is recovered. 

For this vision system, the laser line provides the reference position to determine the radial distortion. In this way, the laser line coordinates (x*_i_*_,*j*_, y*_i_*_,*j*_) are computed via Equations (16) and (17). Based on distorted (x*_i_*_,*j*_, y*_i_*_,*j*_) coordinates, the undistorted coordinates are defined by the following expressions: x*_i_*_,*j*_ = x*_i_*_,*j*_ *+* δx*_i_* and y*_i_*_,*j*_ *=* y*_i_*_,*j*_ *+* δy*_j_,* where (δx*_i_*, δy*_j_*) are the distortions in the *x*-direction and *y*-direction, respectively. Therefore, the expression *S_i_*_,*j*_ = x_1,*j*_ − x*_i_*_,*j*_ represents a distorted line shifting and *s_i_*_,*j*_ = (x_1,*j*_ + δx_1_) − (x*_i_*_,*j*_ + δx*_i_*) provides an undistorted line shifting. In this way, δx*_i_* = (x_1,*j*_ − x*_i_*_,*j*_) − *s_i_*_,*j*_ + δx_1_ = *S_i_*_,*j*_ − *s_i_*_,*j*_ + δx_1_ computes the distortion in the *x*-axis. To obtain the first line shifting without distortion, the laser line is placed near to the image center to achieve δx_1_ = 0, and *s*_1,*j*_ = *S*_1,*j*_. Thus, the expression *s_i_*_,*j*_ = *i* * *S*_1,*j*_ provides the undistorted shifting, and the distortion in the *x*-axis is computed by the expression δx*_i_* = (x_1,*j*_ − x*_i_*_,*j*_) − *i* * S_1,*j*_. The distortion in the *y*-axis is deduced from the expressions (y*_i_*_,1_ − y*_i_*_,*j*_) = (y*_i_*_,1_ + y_1_) − (y*_i_*_,*j*_ + y*_j_*) and *T_i_*_,*j*_ = (y*_i_*_,1_ − y*_i_*_,*j*_). From these terms, the expression δy*_j_* = (y*_i_*_,1_ − y*_i_*_,*j*_) − *j* * *T_i_*_,1_ is obtained to compute the distortion in the *y*-axis.

## 3. Last Lower-Surface Adjustment via Metaheuristic Algorithm

**Figure 4:**
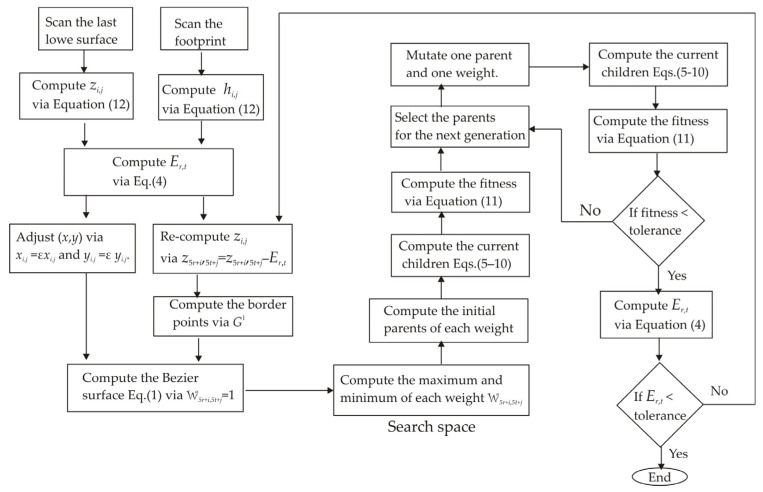
Graphical summary of the methodology to perform the adjusted last lower surface.

**Figure 5:**
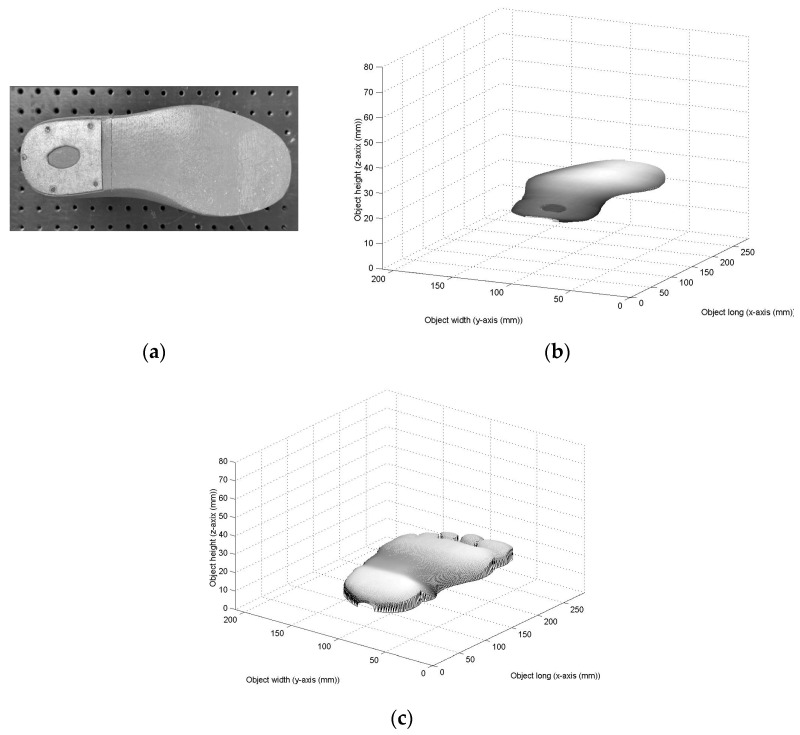
(**a**) Last lower surface to perform the adjusted Bezier surface model. (**b**) Surface generated by the initial Bezier surface model to perform the adjusted last lower surface. (**c**) Footprint topography recovered via laser line scanning.

To perform the adjustment of the last lower-surface model, a Bezier surface model is constructed through the surface topography. The technique to perform adjustment of the last lower surface is carried out based on the graphical summary shown in Figure 4. This graphical summary indicates the steps of the methodology to compute the adjustment of the last lower surface. In this way, the last lower surface is scanned by the vision system shown in Figure 3a, to retrieve the surface contour. The last lower surface to be contoured is shown in Figure 5a. In this way, the laser line is projected on the last lower surface to compute the surface contour via image processing. To do so, the laser line position (x*_i_*_,*j*_, y*_i_*_,*j*_) is computed via Equations (16) and (17) during the scanning. Also, the coordinates (*z_i_*_,*j*_, *y_i_*_,*j*_) are determined by computing Equations (12) and (13), respectively, but the coordinate *x_i_*_,*j*_, is given by the slider device. In this case, 224 laser lines were processed to compute the last lower surface. Thus, the last lower-surface contour is recovered. The accuracy of the recovered surface is determined via relative error by means of the expression

(18) $$Error\%=\frac{100}{n\cdot m}\sum_{i=0}^{n}\sum_{j=0}^{m}\frac{\left|z_{i,j}-H_{i,j}\right|}{H_{i,j}}$$

For this equation, *z_i_*_,*j*_ is the surface computed by Equation (12) via laser line projection, *H_i_*_,*j*_ is the surface measured by a contact method, and *n*·*m* is the data number. In this case, the last lower surface was retrieved with a relative error of 0.4158% through the laser line projection. From this surface contour, the last lower-surface model is adjusted to a footprint topography. To do so, the contour of the last lower surface is modified according to footprint topography on the *x*-axis and *y*-axis. In this way, the footprint outside the last lower surface is determined by the expression *Ey* = *y*_s_ − *y*_f_, where *y_f_* is the contour of the footprint and *y*_s_ is the contour of the last lower surface on the *y*-axis. Thus, the last lower contour is magnified based on the scale factor ε = (*y*_s_ + *Ey*)/*y*_s_, where the last lower-surface coordinates (*x_i_*_,*j*_, *y_i_*_,*j*_) are re-computed by the expressions *x_i_*_,*j*_ = ε*x_i_*_,*j*_ and *y_i_*_,*j*_ = ε *y_i_*_,*j*_. 

**Figure 6:**
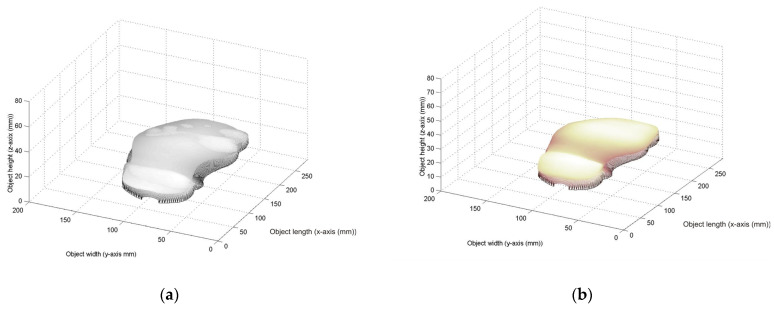
(**a**) Initial last lower surface overlapped on the footprint topography. (**b**) Adjustment of the last lower-surface model to the footprint topography.

Then, a 5th-order Bezier surface model is built from the last lower surface. In this way, the surface is divided into 6 × 6 segments to construct the surfaces *S_r_*_,*t*_(*u*,*v*) via 5th-order Bezier basis functions. Thus, the initial Bezier surface is generated by replacing the control point *P*_5*r*+*i*,5*t*+*j*_ = *z*_5*r*+*i*,5*t*+*j*_ in Equation (1), where the weights are W_5*r*+*i*,5*t*+*j*_ = 1. From this procedure, the initial Bezier surface is generated, and it is shown in Figure 5b, where the scale is in millimeters. Based on this initial Bezier surface, the last lower-surface model is adjusted to a footprint topography through the genetic algorithm. To do so, a footprint is scanned via laser line projection by means of the vision system shown in Figure 3a. In this procedure, the laser line position (x*_i_*_,*j*_, y*_i_*_,*j*_) is computed via Equations (16) and (17), and the coordinates (*z_i_*_,*j*_, *y_i_*_,*j*_) are computed via Equations (12) and Equation (13), respectively. The coordinate *x_i_*_,*j*_, is given by the slider device. Thus, the footprint topography shown in Figure 5c is obtained, where the scale is in millimeters. Then, the metaheuristic genetic algorithm optimizes the control points *P*_5*r*+*i*,5*t*+*j*_ to perform the adjustment of the Bezier surface model to the footprint topography shown in Figure 5c. This procedure optimizes the weights W_5*r*+*i*,5*t*+*j*_ through the five steps described in Section 2.1. In this way, the first step generates the initial population based on the last lower surface and the footprint topography. To carry it out, the initial Bezier surface in Figure 5b is overlapped onto the footprint topography in Figure 5c. The result of this overlapping is shown in Figure 6a. Then, the last lower surface *z_i_*_,*j*_ is moved toward the footprint topography. To do so, the expression *z*_5*r*+*i*,5*t*+*j*_ = *z*_5*r*+*i*,5*t*+*j*_ − *E_r_*_,_*_t_* is computed by means of *E_r_*_,*t*_. Thus, if *E_r_*_,*t*_ > 0, the surface *z*_5*r*+i,5*t*+j_ is moved down. But, if *E_r_*_,*t*_ < 0, the surface *z*_5*r*+i,5*t*+j_ is moved up. Also, the border surface points (*z*_5*r*+5, 5*t*+1_, *z*_5*r*+5, 5*t*+2_, *z*_5*r*+5, 5*t*+3_, *z*_5*r*+5, 5*t*+4_) and (*z*_5*r*+1,5*t*+5_, *z*_5*r*+2,5*t*+5_, *z*_5*r*+3,5*t*+5_, *z*_5*r*+4,5*t*+5_) are determined by the expressions *z*_5*r+5,*j*_ = (*z*_5*r+3,*j*_ + *z*_5*r+6,*j*_)/2 and *z_i_*_,5*t_ = *(z_i_*_,5*t+4_ + *z_i_*_,5*t+6_)/2 to obtain continuity *G*^1^. Then, the maximum and minimum of each weight are computed. To carry it out, the Bezier surface *S_r_*_,*t*_(*u_i_*_,*j*_, *v_i_*_,*j*_) is computed from the moved surface points, where the weights W_5*r*+*i*,5*t*+*j*_ = 1 and the control points *P*_5*r*+*i*,5*t*+*j*_ = *z*_5*r*+*i*,5*t*+*j*_ are substituted in Equation (1). Thus, if the Bezier surface *S_r_*_,*t*_(*u_i_*_,*j*_, *v_i_*_,*j*_) is over the surface *z*_5*r*+*i*,5*t*+*j*_, the maximum is equal to 1, and the minimum is given by the expression [*z*_5*r*+*i*,5*t*+*j*_ − 3*abs(*z*_5*r*+*i*,5*t*+*j*_ − *h*_5*r*+*i*,5*t*+*j*_)]/*z*_5*r*+*i*,5*t*+*j*_. But, if the surface *S_r_*_,*t*_(*u_i_*_,*j*_, *v_i_*_,*j*_) is under *z*_5*r*+*i*,5*t*+*j*_, the minimum is equal to 1 and the maximum is calculated by the expression *z*_5*r*+*i*,5*t*+*j*_ + 3*abs(*z*_5*r*+*i*,5*t*+*j*_ − *h*_5*r*+*i*,5*t*+*j*_)]/*z*_5*r*+*i*,5*t*+*j*_. Thus, the search space has been deduced for each weight. Then, four values are randomly taken from the search space. These values are defined as the parents (P_1,*k*_, P_2,*k*_, P_3,*k*_, and P_4,*k*_), which represent the initial population. Thus, the Bezier surface is moved toward the footprint topography by means of the mean error function and the initial population.

Next, the second step generates the current children by means of explorations and exploitations, where, the children (C_1,*k*_, C_2,*k*_, C_4,*k*_, C_4,*k*_, C_5,*k*_, and C_6,*k*_) are computed via crossover by means of Equations (5)–(10). Then, the third step computes the fitness of parents and children via Equation (11) by employing *S_r_*_,t_(*u_i_*_,*j*_, *v_i_*_,*j*_) and *z*_5*r*+*i*,5*t*+*j*_. Then, the fourth step chooses the (*k* + 1)-generation parents from the best current parents and children: P_1,*k*+1_, is selected from (P_1,*k*_, P_2,*k*_), P_3,*k*+1_ is collected from (P_3,*k*_, P_4,*k*_), P_2,*k*+1_ is taken from (C_1,*k*_, C_2,*k*_, C_3,*k*_) and P_4,*k*+1_ is chosen from (C_4,*k*_, C_5,*k*_, C_6,*k*_). The fifth step replaces the worst parent by a new parent to compute the fitness Equation (11). Thus, if the new parent improves the fitness, the worst parent is mutated. Otherwise, the mutation is not carried out. Also, a parent is randomly designated to mutate a weight, which is chosen in random form, where a new weight replaces the selected weight to calculate the fitness Equation (11). Thus, if the fitness is improved, the weight is mutated. Otherwise, the mutation is not carried out. Then, the second step generates the (*k* + 1)-generation children by computing Equations (5)–(10). Also, the fitness of the children is computed via Equation (11). The procedure to compute the (*k* + 1)-generation population is repeated until the objective function in Equation (11) is minimized for all Bezier surfaces *S_r_*_,*t*_(*u_i_*_,*j*_, *v_i_*_,*j*_). Thus, the optimal weights are obtained to compute the control points *P*_5*r*+*i*,5*t*+*j*_ = W_5*r*+*i*,5*t*+*j*_ (*z*_5*r*+*i*,5*t*+*j*_) for each Bezier surface. Also, the control points (*P*_5*r*+5,5*t*+1_, *P*_5*r*+5,5*t*+2_, *P*_5*r*+5,5*t*+3_, and *P*_5*r*+5,5*t*+4_) and (*P*_5*r*+1,5*t*+5_, *P*_5*r*+2,5*t*+5_, *P*_5*r*+3,5*t*+5_, and *P*_5*r*+4,5*t*+5_) are determined by the expressions *P*_5*r+5,*j*_ = (*P*_5*r+3,*j*_ + *P*_5*r+6,*j*_)/2 and *P_i_*_,5*t_ = *(P_i_*_,5*t+4_ + *P_i_*_,5*t+6_)/2 to obtain continuity *G*^1^. Thus, the Bezier surfaces *S*_0,1_(*u*,*v*), *S*_1,0_(*u*,*v*), …, *S_N_*_,*M*_(*u*,*v*) are determined to accomplish the Bezier surface model. Then, the absolute value of the mean error in Equation (4) between the Bezier surface and the footprint topography is computed for *r* = 0, 1, 2, 3, …, *N* and *t* = 1, 2, 3, …, *M*. Thus, if the absolute value of the mean error is greater than a tolerance, the procedure from step one to step five is repeated to modify the surface model. But, if the error is smaller than the tolerance, the metaheuristic algorithm has finished. By performing 32 modifications, the Bezier surface model has been adjusted. The adjusted last lower surface provided by the metaheuristic algorithm via the Bezier surface is shown in Figure 6b. For this surface model, the metaheuristic algorithm provides a running time of 116 generations to optimize the control points of the adjusted last lower-surface model. In this way, the adjusted last lower-surface model has been optimized through the metaheuristic algorithm. The efficiency of the metaheuristic algorithm is described based on the adjustment of the last lower surface shown to the footprint topography, where the expression *z*_5*r*+*i*,5*t*+*j*_ = *z*_5*r*+*i*,5*t*+*j*_ − *E_r_*_,*t*_ moves the Bezier surface model toward the footprint topography by means of means error *E_r_*_,*t*_. This procedure is accomplished by means of the initial population. Additionally, the metaheuristic algorithm structure provides an efficient procedure to optimize the adjusted Bezier surface model. For instance, the population is determined by means of the search space, which is deduced from the initial Bezier surface model of the last lower surface. This leads to obtaining the adjusted last lower-surface model in a moderated number of iterations. Moreover, the crossover is performed when the average fitness of the parents is improved. In this case, the result of the probability of crossover is in the interval from 0.19 to 0.54.

Also, the mutation is carried out when a new parent improves the fitness. In the same way, the mutation is performed when a new weight improves the fitness. In this case, the mutation probability is established in the interval from 0.22 to 0.56. Based on these statements, the genetic algorithm provides the crossover probability and mutation probability established for surface model optimization. The quality gap is determined by means of the relative error Equation (18), which is computed through the adjusted last lower surface *S_r_*_,*t*_(*u_i_*_,*j*_, *v_i_*_,*j*_) and the footprint topography *h*_5*r*+*i*,5*t*+*j*_. In this case, the metaheuristic algorithm provides a relative error of 4.7%. This means a good adjustment of the last lower-surface model to the footprint topography. 

## 4. Discussion

**Figure 7:**
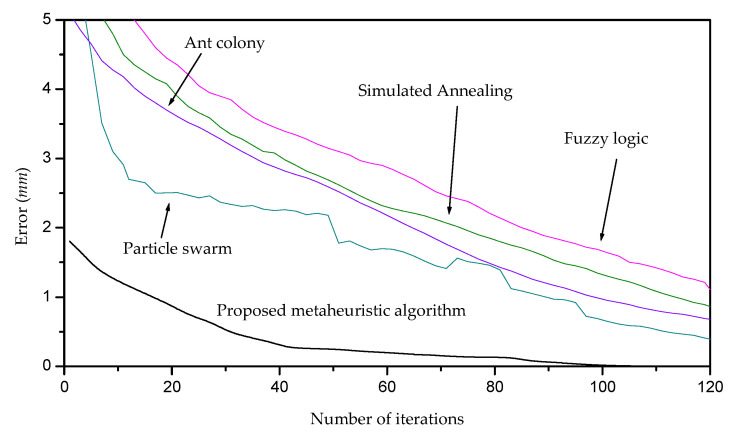
Evolution of the accuracy of the metaheuristic algorithm according to the number of iterations.

The viability of the metaheuristic algorithm is established based on the capability of the adjustment of the last lower-surface model toward the footprint topography [32]. Therefore, the contribution of the adjusted last lower-surface model via the metaheuristic algorithm is determined based on the good adjustment. This statement includes the surface model adjustment and the algorithm efficiency. In these matters, the proposed metaheuristic algorithm provides an accurate last lower-surface model, which fits to the footprint topography in an efficient form. The accuracy of the last lower-surface model adjustment is determined by means of the quality gap, which is calculated through the relative error Equation (18). In this way, the adjustment is achieved by moving the Bezier surface toward the footprint topography by means of the expression *z*_5*r*+*i*,5*t*+*j*_ = *z*_5*r*+*i*,5*t*+*j*_ − *E_r_*_,*t*_. Thus, the metaheuristic algorithm adjusts the last lower-surface model to the footprint topography with a relative error of 4.7%. This relative error is computed via Equation (18), where the surface *z*_5*r*+*i*,5*t*+*j*_ is replaced by *S_r_*_,*t*_(*u_i_*_,*j*_, *v_i_*_,*j*_) and *H_i_*_,*j*_ is replaced by the footprint topography *h_i_*_,*j*_. On the other hand, the metaheuristic algorithm efficiency is evaluated by means of the algorithm structure and solution quality. The metheuristic algorithm provides a suitable structure to optimize the adjustment of the last lower-surface model. This is because the metaheuristc algorithm moves the last lower surface points toward the footprint topography to adjust the Bezier surface model. Also, the research space is generated from the target surface to obtain the optimal weights that produce the adjusted last lower surface. In this way, the metaheuristc algorithm provides the initial population and the mean error *E_r_*_,*t*_, which move the Bezier surface toward the footprint topography. Therefore, a good adjustment is achieved from the first generation onward. Thus, a moderated number of iterations achieve the optimization of the adjusted Bezier surface model. This procedure leads to providing a moderated running time. Thus, the metaheuristic algorithm improves adjustment of the last lower-surface model to the footprint topography of the traditional algorithms. It is because the traditional algorithms adjust the last surface model with a relative error over 7.20% [33,34]. This means that the surface modeling through the metaheuristic algorithm improves the adjustment of the last lower-surface model to the target footprint topography. Also, the laser line scanning provides accuracy for the surface model adjustment. It is because laser line projection provides a high accuracy to retrieve the last lower surface. Additionally, algorithms of artificial intelligence have been implemented by contact methods to perform the last lower-surface model [35]. These algorithms optimize the surface model parameters through the traditional search structure [36], where the research space is not obtained from the target surface. Instead, the proposed metaheuristic algorithm generates the research space from the last lower surface and the footprint topography. This procedure provides the initial weights to move the last lower surface near to the footprint topography. Therefore, the metaheuristic algorithm performs the optimization of the surface model by means of the surface data. This procedure leads to providing a low error from the first generation onward. In this way, the metaheuristic algorithm performs a moderated number of iterations to optimize the control points of the surface model. This is a difference in respect to the traditional algorithms, which generate the initial population in random form [37]. Furthermore, the proposed metaheuristic algorithm searches the optimal weights inside and outside parents via explorations and exploitations. This procedure avoids the elimination of the potential candidates, to find the best solution in an efficient form. The contribution of the proposed metaheuristic algorithm is established based on the traditional metaheuristic algorithms such as particle swarm, ant colony, simulated annealing, and fuzzy logic. To elucidate this comparison, the traditional metaheuristic algorithms for performing the last lower-surface modeling are examined. In this way, the quality gap, running time, and suitable structure of the metaheuristic algorithms are mentioned as follows. For instance, the particle swarm optimization performs the adjusted last lower-surface model with a quality gap of 10.32% of error and a running time of 257 generations. In this case, the adjusted surface model is carried out through the surface which is adjusted to the footprint topography. The ant colony optimization performs the adjusted last lower-surface model with a quality gap of 11.47% of error and a running time of 312 generations. The simulated annealing optimization achieves the adjusted last lower-surface model with a quality gap of 12.18% of error and a running time of 289 generations. The fuzzy logic optimization computes the adjusted last lower-surface model with a quality gap of 13.23% of error and a running time of 362 generations. In the same way, these adjusted surface models have been carried out through the adjusted surface. Additionally, the evolution of the error according to the number of the iterations of the metaheuristic algorithm is depicted in Figure 7. In this figure, the particle swarm, ant colony, simulated annealing, and fuzzy logic are included to corroborate the enhancement of accuracy and speed of the proposed metaheuristic algorithm. For instance, the accuracy improvement is achieved because the metaheuristic algorithm provides accurate control points from the search space to minimize the objective function Equation (11). It can be elucidated by the error of the metaheuristic algorithm shown in Figure 7, where the metaheuristic algorithm begins with a low error, and converges in a moderate number of iterations. Instead, the traditional algorithms begin with high error, and a great number of iterations are performed to achieve the convergence. This is due to the fact that the search space is not defined based on the target surface. On the other hand, the metaheuristic algorithm provides a good speed for achieving the convergence. This is because the proposed algorithm can produce greater changes through the mutation without any dependence on the current result. Instead, the traditional algorithms produce changes which depend on the current results [38]. Therefore, the advancement of the convergence is slow, and it produces a slow speed. 

In the case of the algorithm structure, the proposed algorithm provides a suitable structure to achieve the optimization of the adjusted last lower-surface model. This is because the proposed algorithm provides a flexible selection of the potential candidates, and the operations are performed on the surface model. For instance, the proposed algorithm provides an initial population based on surface data, where the search space is deduced based on the last lower surface and the footprint topography. This leads to finding potential candidates to achieve the optimization from the first generations onward. On the other hand, the particle swarm optimization, ant colony, simulated annealing, and fuzzy logic generate the initial population in random form [39,40,41,42]. Therefore, a low number of potential candidates are tested in every generation. This is because the search space is not related to the target surface. Additionally, the traditional algorithms employ additional equations to perform the optimization. The particle swarm computes a position equation and a velocity equation, ant colony and simulated annealing compute a probability function, and fuzzy logic computes a linguistic function. On the other hand, the metaheuristic algorithm does not compute additional equations. This leads to reduce operations in the iterations. Moreover, the traditional algorithms compute the next population to be tested based on the current results [43]. Therefore, the next result depends on the current result, and the convergence becomes slow. This kind of procedure provides a rigid structure. Instead of this, the proposed metaheuristic can mutate one parent or one weight to produce bigger and smaller changes without any dependence. This leads to achieving a flexible procedure for testing a potential candidate through exploration and exploitation. Additionally, the proposed algorithm structure is compared with the particle swarm structure, which is more useful in surface model optimization. For instance, the particle swarm generates the population of each generation by the term *V_i_*(*t* + 1) = *wV_i_*(*t*) + *αR*_1_[*P^b^_g_*(*t*) − *P_i_*(*t*)] + *βR*_2_[*P^b^_i_*(*t*) − *P_i_*(*t*)], where *w* is the inertia weight, *P_i_*(*t* + 1) = *P_i_*(*t*) + *V_i_*(*t*), *t* is the number of iterations, and (*α*, *β*) represent the learning factors [44]. However, *R*_1_ and *R*_2_ are selected in random form in the interval from 0 to 1. In this way, the particle swarm optimization computes five parameters to generate the population in each *k*-generation. However, these additional parameters are not related to the last lower-surface topography. Instead, the metaheuristic algorithm generates the population of each *k*-generation from the last lower surface by means of the parameters *β* and *α*. In this way, the metaheuristic algorithm provides a more suitable optimization structure than the particle swarm. Moreover, the metaheuristic algorithm provides a more efficient structure than the ant colony optimization and simulated annealing optimization [45,46]. This is because these optimization methods compute more variables than the proposed metaheuristic algorithm. Thus, the suitable optimization structure of the metaheuristic algorithm is elucidated by the result of the adjusted last lower surface, which is shown in Figure 6b. The efficiency of the metaheuristic algorithm is achieved by means of the good adjustment of the last lower surface to the footprint topography, where the Bezier surface model is adjusted to generate a surface that fits the footprint topography. Additionally, the efficiency is elucidated by means of the parameters of the metaheuristic algorithm, where the search space enables the population to construct a surface model near to the optimal surface. This procedure leads to reducing the number of iterations to construct the adjusted last lower-surface model. From these statements, the contribution of the adjustment of last lower-surface model via the metaheuristic algorithm and laser lane projection has been corroborated. 

Moreover, the simple setup enlarges the potentiality of the metaheuristic algorithm to perform the adjustment of the surface model. This is because the arrangement is constructed by simple components such as laser diode, CCD camera, slider device and a computer. The computer employed to perform the adjusted last lower surface is a PC with 2.4 GHz of velocity. The frame rate of the camera is 64 fps. The slider device moves the vision system via control software. Each micro laser line image is processed in 0.0062 sec to retrieve the last lower surface. The adjusted last lower-surface model is accomplished in 268.21 s. Thus, the adjustment of the last lower-surface model to the footprint is performed in a good way. 

## 5. Conclusions

A technique to adjust the last lower-surface model to the footprint topography by means of the metaheuristic algorithm has been presented. The metaheuristic algorithm optimization improves the adjustment of the last lower surface by means of the Bezier surface model. This is because the adjusted last lower-surface model is computed by means of the control points, which are deduced from the last lower surface and the footprint topography. This contribution is elucidated through the adjustment accuracy and efficiency to optimize the adjusted last lower-surface model. The enhancement of the adjustment accuracy is achieved by means of the algorithm structure, which moves the last lower surface toward the footprint topography. These statements are corroborated by the results achieved in the adjustment of the last lower-surface model. This includes adjustment accuracy, running time and efficiency. Also, the enhancement of the surface model adjustment is achieved by means of the laser line scanning, which retrieves accurately the footprint topography. Thus, the last lower-surface model is adjusted based on accurate footprint topography. Moreover, the metaheuristic algorithm efficiency is corroborated through the structure, which moves the last lower surface toward the footprint topography. Thus, the metaheuristic algorithm optimization provides a valuable tool to perform the adjusted last lower-surface model in the field of last manufacturing. In this way, the metaheuristic algorithm optimization via the Bezier surface model and laser line scanning has been performed to adjust the last lower-surface model in a good way.

## Figures and Tables

**Table 1:** Surface control points generated via metaheuristic algorithm for the first generation.

P_i,j_	P _1,1_	P _2,1_	P _3,1_	P _4,1_	C _1,1_	C _2,1_	C _3,1_	C _4,1_	C _5,1_	C _6,1_
*P*_1,1_	27.6631	27.6942	27.8012	27.3941	27.6860	27.6712	27.3150	27.6943	27.5010	27.7320
*P*_1,2_	27.2626	27.0576	28.1047	27.2793	27.2087	27.1114	27.0277	27.8881	27.4960	27.7907
*P*_1,3_	26.5554	27.1792	28.3741	28.2896	27.0154	26.7191	26.5410	28.3519	28.3118	28.4720
*P*_1,4_	26.4820	26.8381	28.4118	28.0240	26.7446	26.5754	26.2859	28.3100	28.1258	28.5242
*P*_2,1_	27.5308	27.7513	26.9891	26.6823	27.6935	27.5887	27.0835	26.9086	26.7628	27.6064
*P*_2,2_	27.3844	27.3601	27.5913	26.8560	27.3780	27.3664	26.9126	27.3983	27.0490	27.8097
*P*_2,3_	28.3583	27.3628	27.5642	27.7009	28.0970	27.6241	27.2106	27.6650	27.6001	28.2015
*P*_2,4_	27.6445	27.2725	28.8380	26.6733	27.5469	27.3702	26.6198	28.2698	27.2415	27.9831
*P*_3,1_	27.4728	26.6841	26.3958	26.5373	27.2658	26.8911	26.7648	26.5002	26.4330	27.6988
*P*_3,2_	27.7614	26.0223	28.3057	28.2128	27.3049	26.4788	26.8248	28.2813	28.2372	28.4999
*P*_3,3_	27.6942	27.9179	27.3464	28.0121	27.8592	27.7529	26.6368	27.8373	27.5211	28.5025
*P*_3,4_	27.1917	29.3360	29.0766	27.5495	28.7732	27.7545	26.1338	28.6757	27.9503	28.5235
*P*_4,1_	26.6644	25.4581	28.4788	25.7886	26.3478	25.7748	25.9372	27.7727	26.4947	27.3046
*P*_4,2_	26.7604	25.5230	27.9312	25.8832	26.4356	25.8478	25.9693	27.3936	26.4207	27.2654
*P*_4,3_	27.2411	28.5583	28.0305	26.1894	28.2126	27.5869	26.0801	27.5473	26.6727	27.4968
*P*_4,4_	28.2807	25.0608	24.5530	27.1651	27.4355	25.9059	26.3211	26.4795	25.2386	28.3272
*fitness*	1.6517	1.6546	1.8663	1.7220	1.6823	1.6240	1.5301	1.8333	1.7542	1.8857

**Table 2:** Surface control points generated via metaheuristic algorithm for the second generation.

P _i,j_	P _1,1_	P _2,2_	P _3,2_	P _4,2_	C _1,2_	C _2,2_	C _3,2_	C _4,2_	C _5,2_	C _6,2_	
*P*_1,1_	27.6631	27.3150	27.3941	27.4582	27.5717	27.4064	27.1151	27.4414	27.4110	27.7624	26.7601
*P*_1,2_	27.2626	27.0277	27.2793	27.8708	27.2009	27.0893	26.9217	27.7156	27.4346	28.0716	26.4022
*P*_1,3_	26.5554	26.5410	28.2896	27.2305	26.5516	26.5448	26.3110	28.0116	27.5085	27.9689	26.1003
*P*_1,4_	26.4820	26.2859	28.0240	26.1476	26.4305	26.3374	26.1759	27.5315	26.6401	27.6328	25.6511
*P*_2,1_	27.5308	27.0835	26.6823	28.0726	27.4134	27.2009	26.8352	27.7077	27.0472	28.2668	26.4028
*P*_2,2_	27.3844	26.9126	26.8560	27.6333	27.2605	27.0365	26.7126	27.4293	27.0600	28.1789	26.1635
*P*_2,3_	28.3583	27.2106	27.7009	27.8151	28.0570	27.5118	26.7106	27.7851	27.7309	28.2557	25.8065
*P*_2,4_	27.6445	26.6198	26.6733	25.9186	27.3755	26.8888	26.3198	26.4752	26.1167	27.6246	25.2117
*P*_3,1_	27.4728	26.7648	26.5373	27.1942	27.2870	26.9507	26.5648	27.0218	26.7097	28.0109	25.8140
*P*_3,2_	27.7614	26.8248	28.2128	27.9188	27.5155	27.0706	26.6218	28.1357	27.9960	28.3602	25.5962
*P*_3,3_	27.6942	26.6368	28.0121	27.3629	27.4166	26.9143	26.0318	27.8417	27.5333	28.1941	25.3564
*P*_3,4_	27.1917	26.1338	27.5495	27.9348	26.9140	26.4115	25.9133	27.8336	27.6506	28.7065	24.6168
*P*_4,1_	26.6644	25.9372	25.7886	26.5850	26.4735	26.1281	25.7371	26.3759	25.9977	27.6829	24.8471
*P*_4,2_	26.7604	25.9693	25.8832	26.7065	26.5528	26.1770	25.8613	26.4904	26.0993	27.6565	24.7543
*P*_4,3_	27.2411	26.0801	26.1894	27.1748	26.9364	26.3849	25.8801	26.9162	26.4481	27.9649	24.6025
*P*_4,4_	28.2807	26.3211	27.1651	24.5809	27.7663	26.8354	26.2221	26.4868	25.2592	27.0996	23.8512
*fitness*	1.6517	1.5301	1.7220	1.6498	1.6194	1.5616	1.0085	1.7388	1.6299	1.8490	0.000461

## Data Availability

The original contributions presented in the study are included in the article, further inquiries can be directed to the corresponding author.
